# SLC4A10 impedes atherosclerosis by diminishing IFN-γ/GZMB levels of CD8^+^ T cells via the MAPK pathway

**DOI:** 10.3389/fimmu.2025.1568999

**Published:** 2025-05-29

**Authors:** Bo Chen, Lei Zhu, Xueguang Lin, Kristine J. S. Kwan, Jie Wang, Yijie Lu, Jialong Li, Ying Deng, Shuai Jiang, Jingdong Tang, Bo Yu

**Affiliations:** ^1^ Department of Vascular Surgery, Shanghai Pudong Hospital, Fudan University Pudong Medical Center, Shanghai, China; ^2^ Shanghai Key Laboratory of Vascular Lesions Regulation and Remodeling, Fudan University Pudong Medical Center, Shanghai, China; ^3^ Department of Vascular Surgery, Huashan Hospital, Fudan University, Shanghai, China

**Keywords:** plaque stability, CD8^+^ T cells, SLC4A10, MAPK pathway, atherosclerosis

## Abstract

**Background and aims:**

CD8^+^ T cell subpopulations participate in the formation of atherosclerotic plaques through activation or exhaustion. Yet, it is unclear which specific subset it critically involved. The SLC4A10^+^ CD8^+^ T cell possess atherogenic attributes and this study aimed to investigate the associated pathway involved in affecting plaque stability.

**Methods:**

Carotid plaques were collected from patients that underwent carotid endarterectomy in our institute and categorized into stable or unstable plaques. The SLC4A10^+^ CD8^+^ T cell subset were investigated. For *in vivo* analysis, carotid artery tangem ligation was performed in 8-week-old, AAV-6 overexpressed mice fed with high-fat diet to acquire unstable carotid plaques. Isolated CD8^+^ T cells were cultivated and their immunopathological characteristics were examined *in vitro*.

**Results:**

SLC4A10^+^ CD8^+^ T cells were significantly enriched in unstable human carotid plaques and were correlated with the apoptosis of vascular smooth muscle cells (VSMCs). SLC4A10-overexpressed mice, serum IL-4, IL-17A, and IL-6 were increased, while the level of granzyme B (GZMB) decreased. The extent of atherosclerotic plaques was mitigated, the amount of collagen fibers were diminished, and the apoptosis of VSMCs were alleviated. Flow cytometry suggested that SLC4A10 decreased the levels of IFN-γ and GZMB in CD8^+^T cells. The CCK8 demonstrated that IFN-γ and GZMB lead to the decrease in MOVAS cell viability. KEGG analysis revealed that SLC4A10^+^ CD8^+^ T cells participated in the MAPK pathway, cytokine-cytokine receptor interaction, TNF signaling pathway, and cell adhesion molecule pathway. The differential expression of related genes MAPK2K6, ELK4, and MAP3K5 in the MAPK pathway were verified.

**Conclusions:**

These data demonstrate that SLC4A10 mitigates cytotoxicity by decreasing the levels of IFN-γ/GZMB of SLC4A10^+^ CD8^+^ T cells via the MAPK pathway, which impedes plaque progression and aids stabilization.

## 1 Introduction

Stroke is the fifth leading cause of death in the United States. There are nearly 700,000 acute ischemic strokes each year, in which plaque erosion or rupture can trigger acute cardiovascular events and cerebrovascular events (CE) (1, 2). Numerous convincing experimental and clinical data suggest that inflammation is fundamentally involved in the formation of atherosclerosis and the pathophysiology of ischemic events (3–5). The concept that plaque rupture leads to cardiovascular and cerebrovascular events has been widely accepted. Plaque rupture may be related to expansion of lipid-rich necrotic core (LRNC), inflammatory cell infiltration, thinning of fibrotic cap, and neovascularization, but its underlying pathophysiological mechanism remains unclear (6, 7).

The inflammatory infiltrate in atherosclerotic lesions is mainly composed of T cells and macrophages, and CD8^+^ T cells account for more than half of the number of T cells during the whole process of plaque development (8, 9). Previous studies have reported both proatherogenic and antiatherogenic effects of CD8^+^ T cells, highlighting the complex role of T cells in atherogenesis (10). Single-cell RNA sequencing (scRNA-Seq) studies have revealed a high diversity of T cells in human atherosclerotic plaques (11, 12). A study also found that SLC4A10^+^ CD8^+^ T cell subset was associated with a high risk of CE in scRNA-Seq analysis and the Biobank of Karolinska Endarterectomy (BIKE) (13). These studies indicated that CD8^+^ T Cells may played an important role in the pathogenesis of atherosclerosis.

In this study, we primarily focus on the SLC4A10^+^ CD8^+^ T cells association in carotid plaques. We further demonstrate that this subset shows a reduction in IFN-γ/GZMB of via the MAPK pathway, which promotes stability in plaques.

## 2 Materials and methods

### 2.1 Human carotid plaque samples

Carotid plaque specimens were obtained from 10 patients who underwent carotid endarterectomy at the Fudan University Pudong Medical Center between January 2023 and July 2023. Indications for carotid endarterectomy (CEA) were based on NASCET and ACAS criteria (n= 10) (14, 15). The specimens acquired by CEA comprise the carotid artery plaque and the intima of the artery, excluding the media and adventitia. The Institutional Ethical Review Boards of Fudan University Pudong Medical Center approved this research(No. QWJWXK-24). All participants gave their written informed consent before surgery. In addition, patients were divided into a stable group and a vulnerable group based on the following standards as described in previous research (16).

### 2.2 Mice and unstable plaque model

ApoE^−/−^ mice were purchased from Nanjing Junke Bioengineering Co., Ltd. (Nanjing, Jiangsu, China) and bred in-house. Animals were kept under standard laboratory conditions; food and water were provided ad libitum. The Institutional Ethical Review Boards of Fudan University Pudong Medical Center approved this research (2023-D-QWJWRC-04). These mice were kept on a standard chow diet for 4 weeks. At week 4, mice were switched to a Western diet containing 20% fat, 1.25% cholesterol, and 0.5% sodium cholate. At week 8, all mice (*n* = 30) were randomly distributed into the control group (*n* = 15) or SLC4A10 overexpression group (*n* = 15). The control group and SLC4A10 overexpression group were injected with 10 vg AAV type 6: AAV-NC or AAV-SLC4A10 diluted in 100 ul phosphate buffered salin, respectively, via tail vein (Generated by HanBio, Inc., Shanghai, China).

The mice model with unstable carotid plaque was generated by tandem stenosis reported previously (17). Briefly, mice were anesthetized with isoflurane (4% for induction, 2% isoflurane for maintenance), and the left common carotid artery and bifurcation were separated. The stenosis diameter is defined by the 0.15mm copper wire applied as a space holder. The first knot was tied under the carotid bifurcation by 1 mm with 7–0 Polypropylene. Subsequently, the second knot was tied at 5 mm below the first knot, Finally, the incision was cleaned with normal saline, and the skin was sutured.

In the 14th week, all mice were sacrificed after anaesthetization, and blood samples and tissue samples were taken for further study.

### 2.3 MACS isolation of CD8^+^ T cells

CD8^+^ T cell isolation kit (Miltenyi Biotec.) containing the biotinylated antibodies cocktail (CD4, CD11b, CD45R, DX5, and Ter119a) was used to negatively isolate CD8^+^ T cells from splenocytes. Spleens of AAV-injected and control mice were disrupted by frosted ends of glass slides, and erythrocytes were lysed to make single-cell suspensions. After incubation with a biotinylated antibody cocktail, anti-biotin magnetic microbeads were added to the cell suspension. Unlabeled CD8^+^ T cells were negatively sorted with a MACS sorter for subsequent use.

### 2.4 Western blot assays

Total protein was isolated from the human plaques, mouse cells, and arteries using RIPA protein lysis buffer (BeyotimeBiotechnology, Shanghai, China), and the protein concentrations were measured using a BCA protein assay kit (Epizyme Biomedical Technology Co., Ltd, Shanghai, China) based on the manufacturer’s protocol. Samples were loaded to 10% 1×PAGE gels (Epizyme, Shanghai, China) and separated using gel electrophoresis. Proteins were transferred to Millipore Immobilon PVDF Transfer Membranes (EMD Millipore, Darmstadt, Germany) and blocked with Protein Free Rapid Blocking Buffer (Epizyme, Shanghai, China) for half an hour. Primary antibodies in Universal Antibody Dilution Buffer (Epizyme, Shanghai, China) (Dilution: 1 to 1000) were added to blots and incubated at 4°C overnight. Blots were washed three times with TBST for 10 min and incubated with HRP-conjugated goat anti-rabbit IgG (1 to 5000 in Universal Antibody Dilution Buffer) (Beyotime Biotechnology, Shanghai, China) for two hours at room temperature. Following washing three times with PBST, protein bands were visualized using the Bio-Rad electrochemiluminescence ECL system. A list of antibodies is provided in **Supplementary Table S1**.

### 2.5 RNA extraction and quantitative RT-qPCR

Total RNA was extracted from human plaques, mouse cells and arteries using the RNAiso Plus kit (Takara Biomedical Technology Co., LTD., Beijing) according to the manufacturer’s protocol. Total RNA (1 μg) was reverse transcribed as cDNA using the PrimeScript™ FAST RT reagent Kit with gDNA Eraser(Takara Biomedical Technology Co., Ltd., Beijing, China), according to the manufacturer’s instructions. The produced cDNA was amplified using fluorescent RT-qPCR using SYBR Green Pro Taq HS (Accurate Biology; Hunan, China), following the manufacturer’s protocol. The amplification conditions were 95.0°C for 10 min, followed by 40 cycles of 95.0°C for 15 s and 60.0°C for 60 s. A list of primers is provided in the **Supplementary Table S2**.

### 2.6 Histological analysis of atherosclerotic lesions

Human plaque and mouse arteries were washed using PBS, fixed in 10% formalin for 48 h, embedded in paraffin, and cut into 5-μm-thick sections. Sections were stained with hematoxylin and eosin (HE), Masson trichrome for histological characterization. They were used to assess atherosclerotic lesion size, collagen content, necrotic core area, and apoptotic cells as previously described. Fluorescence colocalization of SLC4A10 and CD8, α-SMA and TUNEL, CD31 and TUNEL, CD68 and TUNEL was performed. The entire aorta of the ascending aorta to the abdominal aorta of the mice was dissected, periaortic fat and blood clots were removed, and oil red o staining was performed. Panoramic scanner PANNORAMIC DESK/MIDI/250/1000(3DHISTECH, Budapest, Hungary) was used for image acquisition.

### 2.7 Bulk-RNA-sequencing

1 μg total RNA was used for the following library preparation. The poly(A) mRNA isolation was performed using Oligo(dT) beads. The mRNA fragmentation was performed using divalent cations and high temperatures. Priming was performed using Random Primers. First-strand cDNA and the second-strand cDNA were synthesized. The purified double-stranded cDNA was then treated to repair both ends and add a dA-tailing in one reaction, followed by a T-A ligation to add adaptors to both ends. Size selection of Adaptor-ligated DNA was then performed using DNA Clean Beads. Each sample was then amplified by PCR using P5 and P7 primers and the PCR products were validated.

Then libraries with different indexes were multiplexed and loaded on an Illumina HiSeq/Illumina Novaseq/MGI2000 instrument for sequencing using a 2x150 paired-end (PE) configuration according to the manufacturer’s instructions.

150 bp nucleotides were used for the paired-end sequencing on an Illumina NovaSeq 6,000. Raw data were first evaluated by Fast QC for quality control Adapter sequences, low-quality reads, and too-short reads were removed with Trim-galore to obtain clean data. In the beginning transcripts in fasta format are converted from known gff annotation files and indexed properly. Then, with the file as a reference gene file, HTSeq (v0.6.1) estimated gene and isoform expression levels from the pair-end clean data. Differential expression analysis used the DESeq2 Bioconductor package, a model based on the negative binomial distribution. The estimates of dispersion and logarithmic fold changes incorporate data-driven prior distributions, Padj of genes was set < = 0.05 to detect differentially expressed ones. GOSeq (v1.34.1) was used to identify Gene Ontology (GO) terms that annotate a list of enriched genes with a significant padj less or equal to 0.05. Top GO was used to plot DAG. KEGG (Kyoto Encyclopedia of Genes and Genomes) is a collection of databases dealing with genomes, biological pathways, diseases, drugs, and chemical substances (http://en.wikipedia.org/wiki/KEGG). We used scripts in-house to enrich significant differential expression genes in KEGG pathways.

### 2.8 Enzyme-linked immunosorbent assay

ABplex Mouse 7-Plex Custom Panel (ABclonal, Wuhan, China) was used to detect the expression levels of lipid and immune markers in plasma. According to the manufacturer’s instructions, plasma samples were diluted with a sample dilution buffer. 50ul of plasma samples were added to each well, and 50ul of standards of each gradient were reserved for 7 Wells. Then 5ul of microsphere suspension was added to each well, and incubated at 37°C for 1 hour before washing once by magnetic force. 50ul of antibody was added and incubated at 37°C for 0.5 h before washing once. 50ul of fluorescein solution was added and incubated at 37°C for 15 min. Finally, 70ul of washing buffer was added and detected by ABplex-100(ABclonal, Wuhan, China).

### 2.8 Flow cytometry

Approximately 40,000 CD8^+^ T cells were stained with the appropriate antibodies in PBS. For intracellular staining, cells were fixed and permeabilized by using an intracellular staining kit (eBioscience) according to the manufacturer’s protocol. To detect cytokine production, in complete RPMI as stated above at 37°C and 5% CO_2,_ cells were stimulated with eBioscience™ Cell Stimulation Cocktail (Invitrogen, US) for 10 hours, eBioscience™ Protein Transport Inhibitor Cocktail (Invitrogen, US) was added to the co-culture for 6 hours before flow cytometry analysis. Live SLC4A10+CD8+ T cells were gated. Flow cytometry analyses were performed on NOVO Express. A list of antibodies is provided in **Supplementary Table S1**.

### 2.9 Cell viability assay

Cells were seeded in 96-well plates at a density of approximately 1.5 × 10³ cells per well. Once the cells adhered to the wells, GMZB (10 μg/mL) or IFN-γ (100 ng/mL) was separately added to each experimental well for intervention. At each designated time point for detection, the original culture medium was discarded, and the wells were washed twice with PBS. Negative and blank controls were established, with three replicates set for each. 10 μL of CCK-8 working solution was added to each well of the 96-well plate, taking care to avoid the generation of bubbles. Subsequently, the plate was placed in an incubator for 1 hour. After the incubation was completed, the absorbance at 450 nm was determined using a microplate reader.

### 2.10 Statistical analysis

Data are presented as mean ± SEM, and the number of animals in each group is stated in the text. Data were tested for normal distribution and analyzed by using a two-tailed Student’s T-test, Mann-Whitney test, one-way ANOVA, or two-way ANOVA, as appropriate. Spearman correlation coefficient is used for correlation analysis. Statistical analysis was performed by using Prism 10 (GraphPad Software, LLC). Probability values of p < 0.05 were considered significant.

## 3 Results

### 3.1 SLC4A10^+^ CD8^+^ T cells were abundant in unstable atherosclerotic plaques

**Figure 1:**
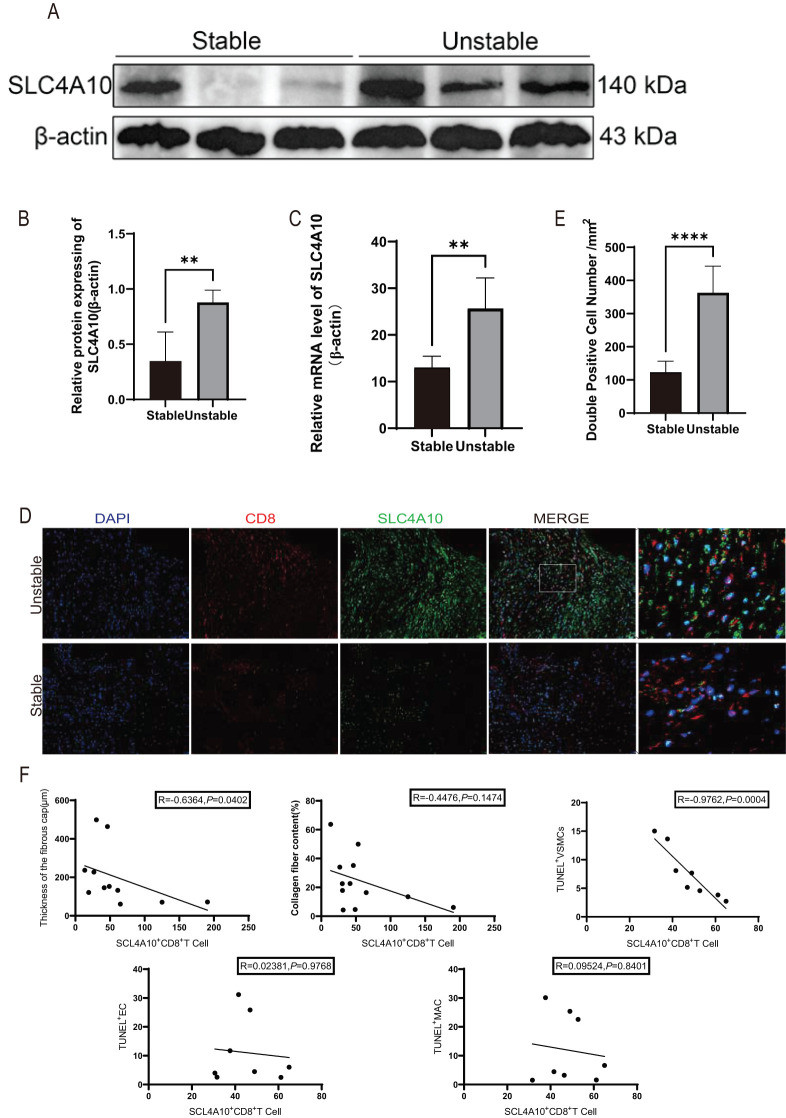
The study of human carotid plaque samples discloses the relationship between SLC4A10^+^CD8^+^T cells and plaque stability. **(A, B)** Western blot analysis was performed to evaluate SLC4A10 expression in unstable and stable human carotid plaques. Quantitative protein expression levels were normalized to β-actin. (N = 6 for each group, *p* < 0.01). **(C)** Graphical representation of RT-qPCR results for SLC4A10 in human carotid plaques. (N = 6, *p* < 0.01). **(D, E)** Representative images and quantification of SLC4A10 and CD8 immunofluorescence co-staining in human carotid plaques. (N = 6 for each group, *p* < 0.0001). Scale bar = 100 μm. **(F)** The scatter plot of Spearman correlation coefficients analyzing the relationship between SLC4A10^+^ CD8^+^ T cells and the thickness of plaque fibrous cap (R = -0.6364, *p* = 0.0402), collagen fiber content (R-0.4476, *p* = 0.1474), TUNE^+^ SMC (R = -0.9762, *p* = 0.0004), TUNEL^+^ endothelial cells (R = 0.02381, *p* = 0.9768), and TUNEL^+^ macrophages (R = 0.09524, *p* = 0.8401). (**: p<0.01; ****: p<0.0001).

Our previous investigations have indicated a heightened risk of CE associated with SLC4A10^+^ CD8^+^ T cells. Herein, we utilized human carotid artery specimens obtained after clinical CEA to investigate the correlation between SLC4A10^+^ CD8^+^ T cells and plaque stability. We found that a higher protein expression of SLC4A10 in unstable plaques compared to stable plaques (*p* < 0.01) (**Figures 1A, B**). The mRNA level of SLC4A10 was also significantly higher in unstable than in stable plaques (*p* < 0.01) (**Figure 1C**). Co-immunofluorescence staining results revealed a markedly higher (*p* < 0.0001) abundance of SLC4A10^+^ CD8^+^ T cells within unstable plaques compared to stable ones (**Figures 1D, E**). The unstable behavior of plaques is associated with the thickness of the fibrous cap, the content of collagen fibers, and the quantity of apoptotic cells. A further analysis was carried out regarding the relationship between the number of SLC4A10^+^ CD8^+^ T cells and the unstable behavior of plaques. The results demonstrated that the number of SLC4A10^+^ CD8^+^ T cells was negatively correlated with the thickness of the fibrous cap (R = -0.6364, *p* < 0.05) and the quantity of apoptotic VSMCs (R = -0.9762, *p* < 0.001), and there was no correlation with the content of collagen fibers, the number of apoptotic endothelial cells, and the number of apoptotic macrophages (**Figure 1F**).

### 3.2 The overexpression of SLC4A10 in CD8^+^ T cells elevates the level of inflammation and reduces cytotoxicity

**Figure 2:**
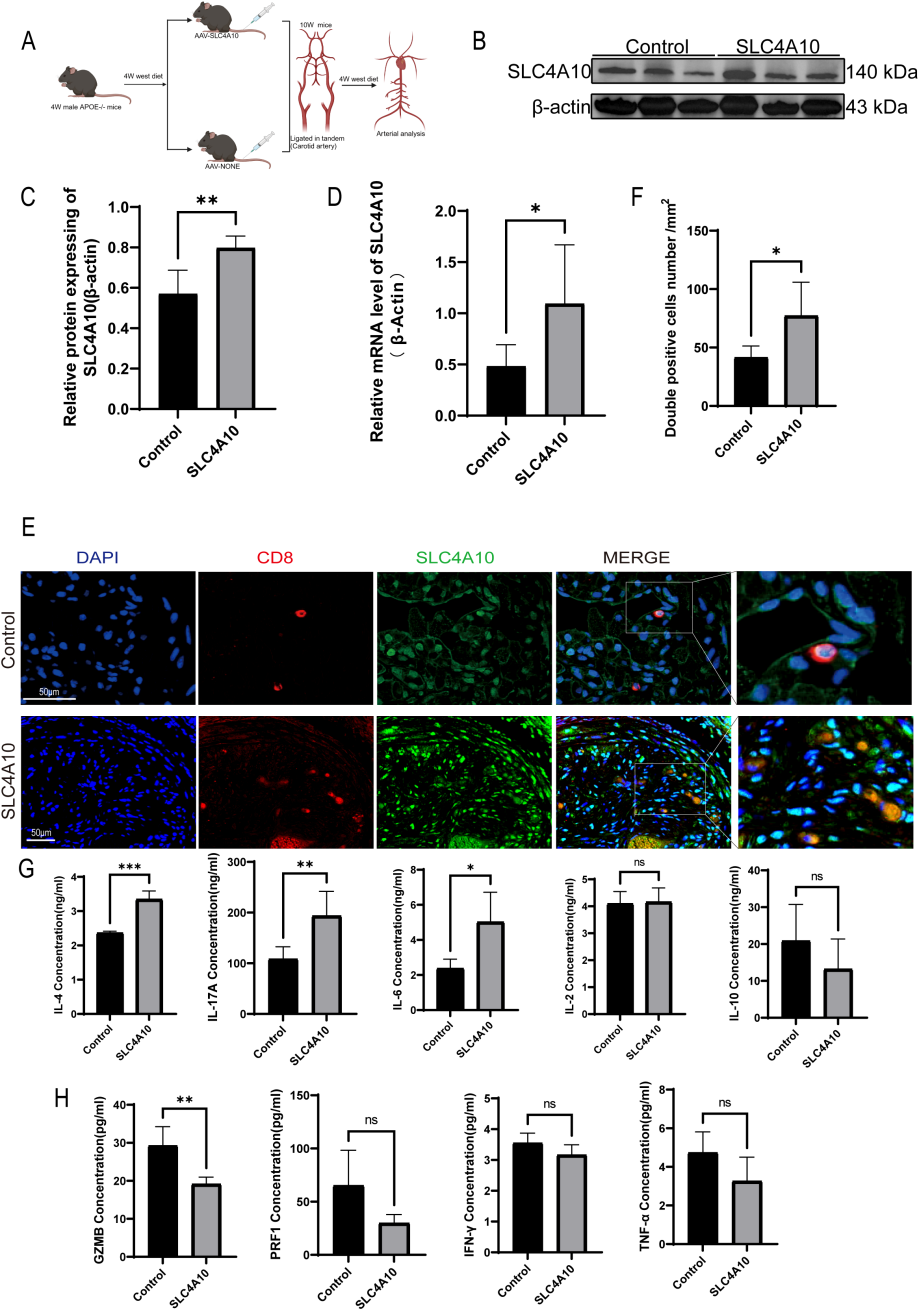
AAV-mediated overexpression of SLC4A10 augmented the quantity of SLC4A10^+^CD8^+^T cells in the carotid plaques of mice and elevated the inflammatory level in the serum. **(A)** Schematic representation of the experimental design. **(B, C)** Western blot analysis of SLC4A10 expression in carotid plaques from AAV-SLC4A10 and AAV-NONE groups of mice. Quantitative protein expression levels were normalized to β-actin. (N=6 for each group, *p* < 0.05). **(D)** Graphical representation of RT-qPCR results for SLC4A10 in mouse carotid plaques. (N = 6, *p* < 0.01). **(E, F)** Representative images and quantification of SLC4A10 and CD8 immunofluorescence co-staining in mouse carotid plaques. (N = 6 for each group, *p* < 0.01). Scale bar = 50 μm. **(G)** The expression levels of IL-4 (*p* < 0.0001), IL-17A (*p* < 0.01), IL-6 (*p* < 0.05), IL-2 (*p* > 0.05) and IL-10 (*p* > 0.05) in the serum of the overexpression group and the control group were determined by ELISA (N = 5 for each group). **(H)** The expression levels of GZMB (*p* < 0.05), PRF1 (*p* > 0.05), IFN-γ (*p* > 0.05) and TNF-α (*p* > 0.05) in the serum of mice in the overexpression group and the control group were determined by ELISA (N = 5 for each group). (*: p<0.05; **: p<0.01; ***: p<0.001; ns: p>0.05).

The upregulation of SLC4A10^+^ CD8^+^ T cells was significant in unstable plaques. However, the specific role of SLC4A10^+^ CD8^+^ T cells in plaque stability remains unclear. To induce an unstable carotid plaque model, we used high-fat feeding, AAV overexpression, and tandem ligation techniques, as shown in the diagram (**Figure 2A**). The results revealed a significantly higher level of SLC4A10 protein in the arteries of the SLC4A10 overexpression group compared to that of the AAV-NONE group (*p* < 0.01) (**Figures 2B, C**). The results revealed a significantly higher mRNA expression level of SLC4A10 in the overexpression group compared to that in the AAV-NONE group (*p* < 0.05) (**Figure 2D**). The results of immunofluorescence co-staining showed that the number of SLC4A10^+^ CD8^+^ T cells in the overexpression group was significantly increased compared to the AAV-NONE group (*p* < 0.05) (**Figures 2E, F**). The results of serum inflammation level analysis showed that the serum levels of IL-4 (*p* < 0.001), IL-17A (*p* < 0.01), and IL-6 (*p* < 0.05) in the experimental group were significantly higher than those in the AAV-NONE group, and there was no significant difference in IL-2 and IL-10 between the two groups (**Figure 2G**). The results showed that GZMB (*p* < 0.05) was significantly reduced, while the levels of PRF-1, IFN-γ and TNF-α were not significantly different (**Figure 2H**). The results of serum lipid detection revealed that the overexpression of SLC4A10 had no significant impact on the lipid metabolic profile in mice (**Supplementary Figure S1**).

### 3.3 Overexpression of SLC4A10 attenuated atherosclerotic plaque progression in mice

**Figure 3:**
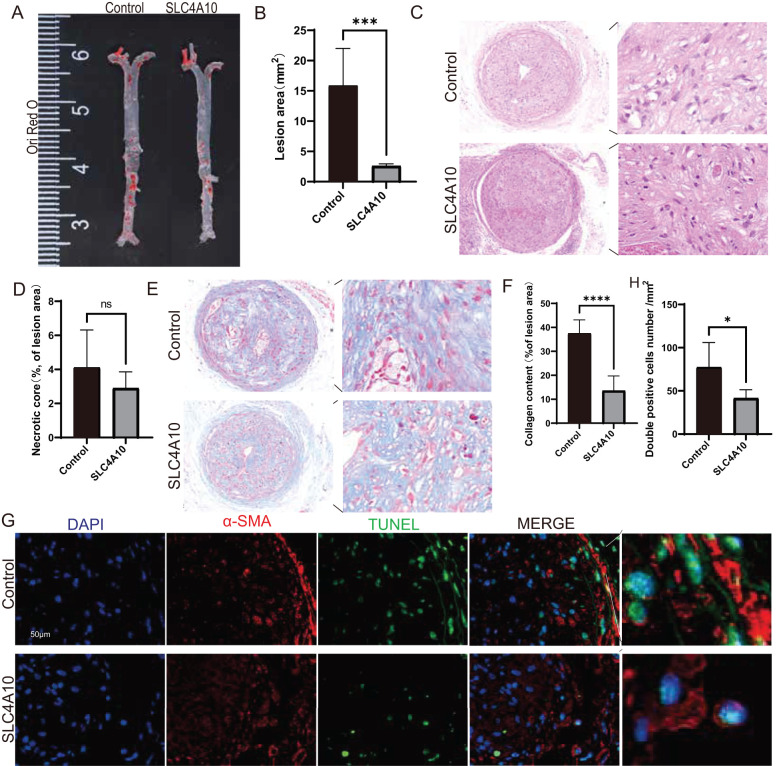
Overexpression of SLC4A10 facilitated the development of atherosclerosis and plaque instability in mice. **(A, B)** Representative en face Oil Red O staining of the aortas of mice from the SLC4A10 and NONE groups. Quantitative assessment of the atherosclerotic plaque area in mice of the SLC4A10 and NONE groups with en face Oil Red O staining (N = 5 for each group, *p* < 0.001). **(C, D)** Representative HE images of carotid plaques in the SLC4A10 group and the NONE group, and quantitative assessment of the necrotic core area. (N = 5 for each group, *p* > 0.05). Scale bar = 50 μm. **(E, F)** Representative Masson images of carotid plaques in mice of the SLC4A10 group and the NONE group, and quantitative assessment of collagen content. (N = 5 for each group, *p* <0.0001) Scale bar = 50 μm. **(G, H)** Representative images and quantification of TUNEL and α-SMA immunofluorescence co-staining in mouse carotid plaques. (N = 5 for each group, *p* < 0.01). Scale bar = 50 μm. (*: p<0.05; ***: p<0.001; ****: p<0.0001; ns: p>0.05).

Our previous study demonstrated that CD8^+^ T cells overexpressing SLC4A10 were able to infiltrate mouse arteries and subsequently examined the atherosclerotic phenotype in mice. Oil red O staining of whole aortic tissue showed a significantly reduced degree of atherosclerosis in the overexpression group compared with the AAV-NONE group (*p < 0.001*) (**Figures 3A, B**). HE staining showed no significant difference in the area of necrotic core within the arterial plaque in the overexpression group compared with the control group (**Figures 3C, D**). The percentage of collagen fibers in the overexpression group was markedly lower than that in the control group (*p < 0.0001*) (**Figures 3E, F**). TUNEL and α-SMA immunofluorescence co-staining showed that the number of apoptotic vascular smooth muscle cells in the arteries of mice in the SLC4A10 overexpression group was significantly reduced (*p* < 0.05) (**Figures 3G, H**).

### 3.4 The gene SLC4A10 is linked to both the inflammatory and cytotoxic phenotypes of CD8^+^ T cells

**Figure 4:**
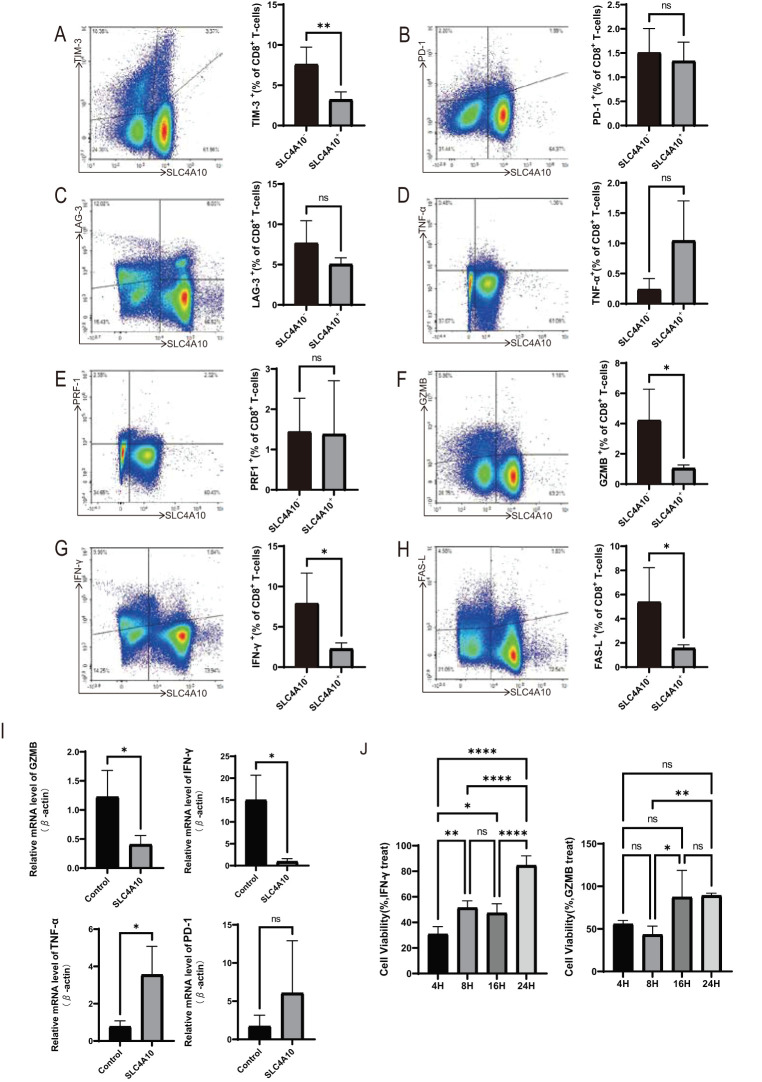
The cytotoxic and pro-inflammatory secretion ability of SLC4A10^+^CD8^+^T cells decreased, and their cytotoxicity to MOVAS cells also decreased. **(A)** Representative images and statistical graphs for the expression of TIM-3 in CD8^+^ T cells analyzed by flow cytometry (N = 4 in each group, *p* < 0.01). **(B)** Representative images and statistical graphs for the expression of PD-1 in CD8^+^ T cells analyzed by flow cytometry (N = 4 in each group, *p* > 0.05). **(C)** Representative images and statistical graphs for the expression of LAG-3 in CD8^+^ T cells analyzed by flow cytometry (N = 4 in each group,*p* > 0.05). **(D)** Representative images and statistical graphs for the expression of TNF-α in CD8^+^ T cells analyzed by flow cytometry (N = 4 in each group, *p* > 0.05). **(E)** Representative images and statistical graphs for the expression of PRF-1 in CD8^+^ T cells analyzed by flow cytometry (N = 4 in each group, *p* > 0.05). **(F)** Representative images and statistical graphs for the expression of GZMB in CD8^+^ T cells analyzed by flow cytometry (N = 4 in each group, *p* < 0.05). **(G)** Representative images and statistical graphs for the expression of IFN-γ in CD8^+^ T cells analyzed by flow cytometry (N = 4 in each group, *p* < 0.05). **(H)** Representative images and statistical graphs for the expression of FAS-L in CD8^+^ T cells analyzed by flow cytometry (N = 4 in each group, *p* < 0.05). **(I)** The RT-qPCR result graph of GZMB (*p* < 0.05), IFN-γ (p < 0.05), TNF-α (*p* < 0.05), PD-1 (*p* > 0.05), in CD8^+^ T cells of the overexpression group versus the control group of mice (N=4 per group). **(J)** Cell viability at different time points after treatment with GZMB and IFN-γ. IFN-γ significantly influenced cell viability at 4 h, 8 h, and 16 h post-intervention, with a diminished effect observed at 24 (h) GZMB exhibited significant inhibition of cell viability at 4 h and 8 h, but this inhibitory effect gradually attenuated at 16 h and 24 h. (*: p<0.05; **: p<0.01; ****: p<0.0001; ns: p>0.05).

Flow cytometry findings indicated that the proportion of TIM-3+ cell subsets within CD8^+^ T cells in the SLC4A10^+^ group was significantly lower than that in the SLC4A10- group (p < 0.01) (**Figure 4A**). No significant disparity was observed in PD-1^+^ and LAG-3^+^ cells between the two groups (p > 0.05) (**Figures 4B, C**). Subsequently, we conducted an analysis of the expression levels of cytokines. The proportion of TNF-α^+^ cells in the SLC4A10^+^ group was higher than that in the control group; however, no significant difference was noted (*p* > 0.05) (**Figure 4D**). There was no marked difference in the PRF1^+^ cell subsets between the two groups (*p* > 0.05) (**Figure 4E**). The subsets of GZMB^+^, INF-γ^+^, and FAS-L^+^ in the SLC4A10^+^ group were significantly lower than those in the control group (**Figures 4G, H**). RT-qPCR results indicated that, compared with the control group, the mRNA levels of GZMB (*p* < 0.05) and IFN-γ (p < 0.05) were significantly decreased, the mRNA level of TNF-α (*p* < 0.05) was significantly increased, and there was no significant difference in PD-1 (*p* > 0.05) (**Figure 4I**).

After 4 h of IFN-γ intervention, cell viability was conspicuously lower than that at 8 h (*p* < 0.01), 16 h (*p* < 0.05), and 24 h (*p* < 0.0001). No significant discrepancy in cell viability was discerned between the 8 h and 16 h intervention groups (*p* > 0.05). At 24 h, cell viability was significantly augmented compared to the 16 h group (*p* < 0.0001), and cell viability exceeded 80%. Following GZMB intervention, there was no significant variance in cell viability between the 4 h and 8 h groups (*p* > 0.05), yet cell viability was merely approximately 50%. In contrast to the 8 h intervention group, cell viability was significantly reduced at 16 h (*p* < 0.05) and 24 h *(p* < 0.01). No significant dissimilarity in cell viability was detected between the 16 h and 24 h groups, and cell viability approximated 70% (**Figure 4J**). Both GZMB and IFN-γ can notably influence the cell viability of VSMCs. IFN-γ influences cell viability in the early and intermediate stages of intervention, while GZMB affects cell viability in the early stage of intervention.

### 3.5 The KEGG analysis indicates that SLC4A10 modulates the function of CD8^+^ T cells via the MAPK pathway

**Figure 5:**
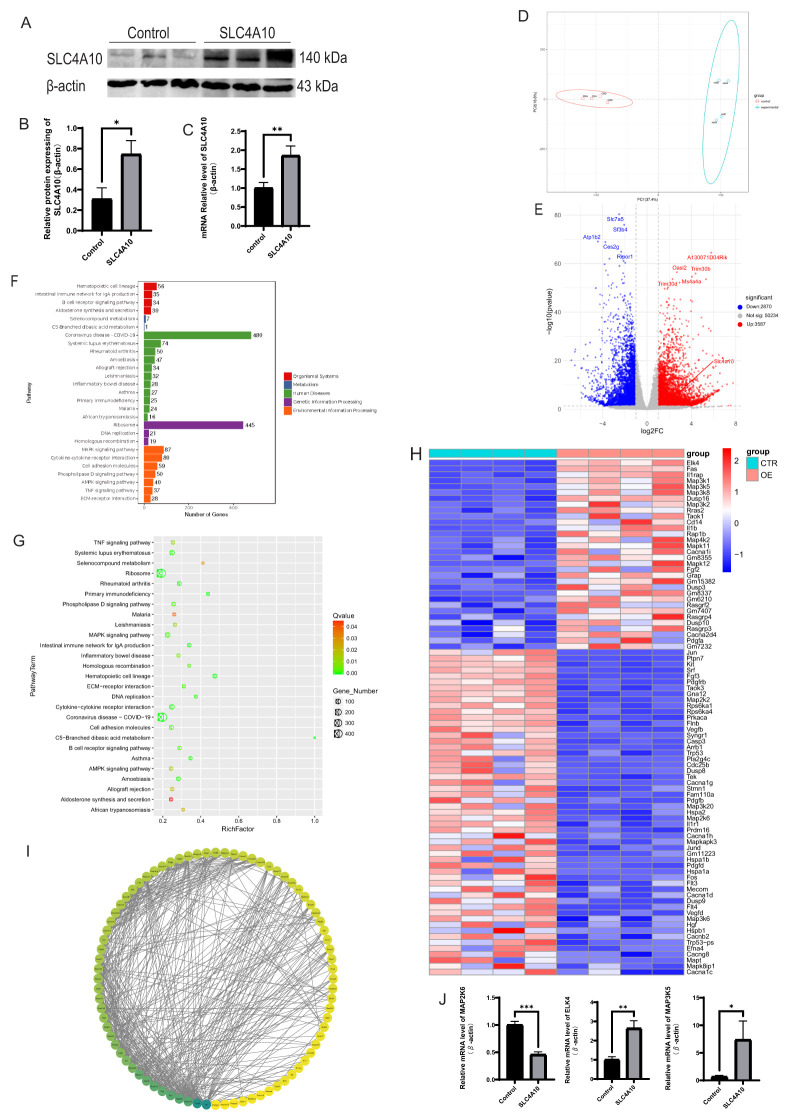
SLC4A10^+^ CD8^+^ T cells are correlated with immunity, inflammatory reactions, and the MAPK pathway. **(A, B)** Western blot analysis of SLC4A10 expression in mouse CD8^+^T cells, with quantitative assessment of protein levels normalized to β-actin. (N=3 for each group, *p* < 0.05). **(C)** Overview of RT-qPCR results for SLC4A10 expression in mouse CD8^+^T cells. (N = 3, *p* < 0.01). **(D)** PCA showed significant heterogeneity between the overexpression and control groups. **(E)** Volcano plot illustrating DE-genes in the SLC4A10 group compared to the Control group, with blue and red representing downregulated and upregulated genes, respectively. **(F)** Bar graph of KEGG pathway enrichment analysis of differential genes. **(G)** Enrichment factor bubble plot for KEGG pathway enrichment analysis of differential genes. **(H)** Heat map of the expression changes of genes involved in the MAPK pathway. **(I)** Diagram of gene interaction analysis of genes involved in MAPK pathway changes. **(J)** The RT-qPCR result graph of MAP2K6 (*p* < 0.01), ELK4 (*p* < 0.05) and MAP3K5 (*p* < 0.05) in CD8^+^T cells of the overexpression group versus the control group of mice (N = 3). (*: p<0.05; **: p<0.01; ***: p<0.001).

Western blot analysis revealed that the SLC4A10 proteins in the overexpression group were significantly higher than that in the control group (*p* < 0.05) (**Figures 5A, B**). In comparison with the control group, the mRNA level of SLC4A10 in the overexpression group was significantly elevated (*p* < 0.01) (**Figure 5C**). Principal component analysis indicated that the overexpression group and the control group were distinctly classified into two disparate clusters (**Figure 5D**). From the volcano plot, a total of 6,457 differentially expressed genes were analyzed, encompassing 2,870 down-regulated genes and 3,587 up-regulated genes. Furthermore, the expression level of SLC4A10 in the overexpression group manifested a significant elevation (**Figure 5E**). KEGG functional analysis demonstrated that the differentially expressed genes were prominently enriched in the MAPK, ribosome, hematopoietic cell lineage, and cytokine-cytokine receptor interaction pathways. Although the enrichment factor levels were not high, the number of differentially expressed genes associated with the MAPK pathway was considerable (**Figures 5F, G**). Further, the alteration levels of differentially expressed genes related to the MAPK pathway were exhibited through a heatmap. The key and downstream factors of the MAPK pathway, such as MAP3K5, MAP2K6, and ELK4, all exhibited differential variations; the expressions of ELK4, FAS, and MAP3K5 were conspicuously increased in the overexpression group, while the expressions of MAP2K6, JUN, and PTPN7 were significantly decreased (**Figure 5H**).

The gene interaction analysis of genes related to MAPK pathway changes included 80 genes, 459 interaction lines, and formed 80 nodes (**Figure 5I**). Some genes were randomly chosen and verified by RT-qPCR to validate the results of RNA-seq. The results showed that compared with the control group, the mRNA levels of MAP2K6 (*p* < 0.001) were significantly decreased, while MAP3K5 (*p* < 0.05) and ELK4 (*p* < 0.01) were significantly increased, which was consistent with the RNA-seq results (**Figure 5J**).

## 4 Discussion

In this study, we investigated the role of SLC4A10^+^ CD8^+^ T lymphocytes in atherogenesis and plaque stability, as well as their underlying molecular mechanism. It was found that the number of SLC4A10^+^ CD8^+^ T cells was significantly increased in unstable plaques and showed a significant negative correlation with the thickness of the fibrous cap and the number of apoptotic smooth muscle cells. AAV-6-induced overexpression of SLC4A10 in CD8^+^ T cells of mice resulted in a decreased severity of atherosclerosis, a reduced content of collagen fibers, and a decreased number of apoptotic VSMCs. SLC4A10 attenuates the cytotoxic effect on VSMCs by reducing the expression of IFN-γ and GZMB. SLC4A10^+^ CD8^+^ T cells were correlated with the MAPK pathway and functions related to ribosomes and hematopoietic cell lines.

Atherosclerosis is a chronic inflammatory disease with autoimmune components that has a close association with the onset and progression of cardiovascular and cerebrovascular diseases (18). The role of T cells and their subsets in the occurrence and progression of atherosclerosis has garnered increasing attention in recent studies. In recent studies, there has been growing interest in the involvement of T cells and their subsets in both the development and advancement of atherosclerosis (19). Previous studies have demonstrated that CD8^+^ T cells constitute more than half of the total number of T cells in the plaques of both humans and mice (20, 21). CD8^+^ T cells induce cell death and promote cell apoptosis via multiple cytotoxic pathways (22–24). The cytotoxicity of CD8^+^ T cells against VSMCs and endothelial cells is involved in the progression of the lesion and the instability of plaques. The depletion of CD8^+^ T cells using antibodies in high-fat fed ApoE^-/-^ mice has been shown by several studies to attenuate atherosclerosis (25–27). CD8^+^ T cells are capable of mediating the atheroprotective effect of APOB-100-related peptide immunity (28). The development in single-cell technology has revealed new subsets of CD8^+^ T cells, such as CD69-expressing ones with reduced cytotoxicity and previously unidentified IFI44L^+^ and TOP2A^+^ subtypes (12, 29). A recent single-cell analytical study indicates that SLC4A10^+^ CD8^+^ T cells may possess pro-inflammatory, polarization, and atherogenic characteristics, and are concurrently associated with the occurrence of cerebrovascular events (13). Firstly, we verified the accumulation of SLC4A10^+^ CD8^+^ T cells in unstable carotid plaques. To further investigate the impact of this novel cell subset on plaque stability, we conducted relevant experiments in mouse models by overexpressing the SLC4A10 gene. Our research on this newly discovered CD8^+^ T cell subset revealed that after overexpressing SLC4A10 in mice, the degree of atherosclerosis was mitigated, the content of collagen fibers was reduced, and the number of apoptotic VSMCs was decreased. It was discovered during the research on carotid artery plaques in humans that the quantity of SLC4A10^+^ CD8^+^ T cells exhibited a significantly negative correlation with the number of apoptotic smooth muscle cells and the thickness of the fibrous cap, and the correlation with the number of apoptotic smooth muscle cells was more pronounced. The results of mouse experiments have indicated that SLC4A10^+^ CD8^+^ T cells may suppress the progression of plaques. There are numerous factors influencing plaque stability. In this study, we only observed the accumulation of SLC4A10^+^ CD8^+^ T cells in unstable plaques through clinical specimens and found that it was correlated with the reduction in the number of apoptotic smooth muscle cells. This result suggests that SLC4A10^+^ CD8^+^ T cells may play a certain role in promoting plaque stability, and their accumulation might be a result of a stress response. Moreover, in mouse experiments, it was also found that the number of SLC4A10^+^ CD8^+^ T cells was negatively correlated with the number of apoptotic smooth muscle cells. This finding further substantiates that SLC4A10^+^ CD8^+^ T cells are elements that attenuate plaque progression and facilitate the stable development of plaques.

Through the analysis of the expression of lipid levels in mouse serum, no obvious differences were detected. Moreover, the research on serum inflammation indicated that after overexpression, the inflammatory levels of IL-4, IL-17A, and IL-6 in mouse serum increased, while the level of cytotoxin GZMB decreased. It has been reported that CD8^+^ T cells deficient in cytotoxic enzymes PRF1 or GZMB and cytokine IFN-γ exhibited reduced pro-atherosclerotic potential (30). CD8^+^ T cells mainly exert significant effects on the progression of atherosclerosis and the unstable development of plaques by releasing cytotoxins and inflammatory cytokines (31). The results of flow cytometry indicated that the positive proportions of IFN-γ, GZMB, and FAS-L in SLC4A10^+^ CD8^+^ T cells were significantly lower than those in the control group, while the positive proportion of TNF-α was higher in the SLC4A10^+^ CD8^+^ T cells but without significance. IFN-γ and GZMB decreased several folds compared with the control group. The proportion of the cytotoxic factor PRF1 showed no significant difference in the two cell types. Additionally, we also investigated the exhaustion phenotype of SLC4A10^+^ CD8^+^ T cells and discovered that the proportion of TIM-3^+^ cells was significantly elevated in SLC4A10^+^ cells, while there was no obvious difference in PD-1 and LAG-3 between the two types of cells. This indicates that the influence of SLC4A10 on the exhaustion phenotype of T cells might not occur via the PD-1 or TIM-3 pathways. In order to study the influence of the decreased levels of IFN-γ and GZMB in SL4A10^+^ cells on smooth muscle cells, a CCK-8 experiment was carried out. IFN-γ mainly influences the viability of cells in the early and middle phases of intervention, significantly affecting cell viability from 4 h to 16 h. GZMB mainly affects the viability of cells in the early phase of intervention, significantly influencing cell viability from 4 h to 8 h. All of this indicates that IFN-γ and GZMB induce SMC apoptosis, promoting atherosclerosis. It has been shown that overexpression of SLC4A10 leads to a reduction in the secretion of GZMB and IFN-γ, thereby attenuating the progression of atherosclerosis and facilitating the stable development of plaques.

NBCN_2_/NCBE, a protein encoded by SLC4A10, functions as a Na^+^-dependent Cl^−^ and HCO3^−^ exchanger while also possessing the ability to self-exchange Cl^−^ ions (32, 33). This transporter is predominantly expressed in the brain, with its highest expression levels observed in the cortical cortex, hippocampus, and cerebellum. Additionally, this transporter can also be found in other tissues such as the stomach and duodenum (34–38). The role of SLC4A10 in CD8^+^ T cells remains unreported, while several studies have identified SLC4A10 as a marker for mucosa-associated resident T cells (39). To study the influence of SLC4A10 on CD8^+^ T cells, we utilized AAV-6 to overexpress SLC4A10 in mouse CD8^+^ T cells and conducted RNA-seq on CD8^+^ T cells isolated from the spleens of the overexpressing mice. KEGG analysis reveals that they are engaged in crucial signaling pathways such as MAPK cytokine-cytokine receptor binding and cell adhesion molecules. It has been discovered that the senescence of CD8+ T cells is mediated by p38 MAPK signalling (40). The transcriptional levels of the key genes MAP3K5 and the downstream significant effector molecule ELK4 in the P38 MAPK pathway were conspicuously higher than those in the control group, while the transcriptional level of another key gene MAP2K6 was markedly lower than that in the control group. The remarkable changes in two key genes and one important effector molecule of the P38 MAPK pathway suggest that SLC4A10 influences the cytotoxic function of CD8^+^ T cells via the MAPK pathway. FAS is a death receptor on the cell surface and pertains to the tumor necrosis factor receptor superfamily. Once Fas combines with its ligand FasL, it is capable of initiating the apoptotic signaling pathway, being one of the crucial mechanisms of apoptosis (41). Among the differentially expressed genes within the MAPK pathway, it was discovered that the overexpression of SLC4A10 also facilitated the expression of FAS, suggesting that the overexpression of SLC4A10 could potentially result in excessive activation or apoptotic disorder of immune cells, thus influencing the cytotoxicity of CD8^+^ T cells. Nevertheless, the specific molecular mechanism through which SLC4A10 functions via the MAPK pathway has not yet been clarified, and this offers a direction for our future studies. To sum up, SLC4A10 might lead to the decline of CD8^+^ T cell viability through the MAPK pathway, resulting in decreased expression levels of IFN-γ and GZMB and the attenuation of cytotoxicity.

We conducted a preliminary exploration of the role of SLC4A10^+^ CD8^+^ T cells in atherosclerosis and plaque stability. Nevertheless, our study does possess certain limitations. We used AAV-6 to achieve overexpression of SLC4A10 in CD8^+^ T cells of mice and preliminarily examined the function of SLC4A10 in the advancement of atherosclerosis and plaque stability. We discovered that SLC4A10 attenuates the generation of plaque collagen fibers; nevertheless, we have not conducted further research on the mechanism by which it diminishes collagen fiber formation, and this represents a future research direction. It was verified that the expressions of MAP3K5 and ELK4 were elevated in SLC4A10^+^ CD8^+^ T cells, yet no further exploration was conducted on the specific molecular mechanisms. We found that SLC4A10 might attenuate the expression of IFN-γ and GZMB via the P38 MAPK pathway. The relationship between the increased proportion of TIM-3 and the exhausted phenotype of CD8^+^ T cells was not deeply investigated.

In summary, this study supports the fact that SLC4A10 weakens the toxic functions of CD8^+^ T cells in secreting IFN-γ and GZMB via the MAPK pathway, exerting a significant role in decelerating the development of atherosclerosis and facilitating plaque stability. Therefore, SLC4A10^+^ CD8^+^ T cells serve as a cell subset with chronically stimulated and elevated responses to inflammation in atherosclerosis, exerting a protective effect on the development of atherosclerosis.

## Data Availability

The raw data supporting the conclusions of this article will be made available by the authors, without undue reservation.

## Supplementary material

Supplementary Figure 1 Statistical analysis of serum HDL, LDL, cholesterol, and triglyceride levels in mice.
