# Supplementary figures and images for: SLC4A10 impedes atherosclerosis by diminishing IFN-γ/GZMB levels of CD8+ T cells via the MAPK pathway
Source: Front Immunol. 2025 May 29;16:1568999. doi: 10.3389/fimmu.2025.1568999 (PMC12159029; doi:10.3389/fimmu.2025.1568999)

A

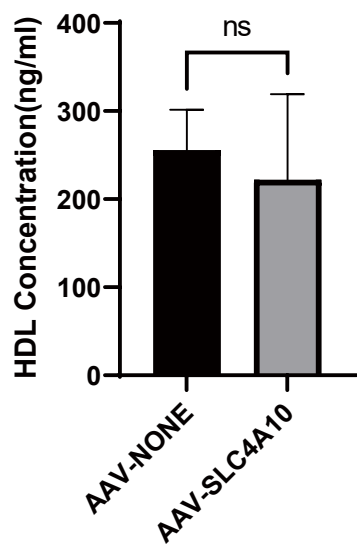

B

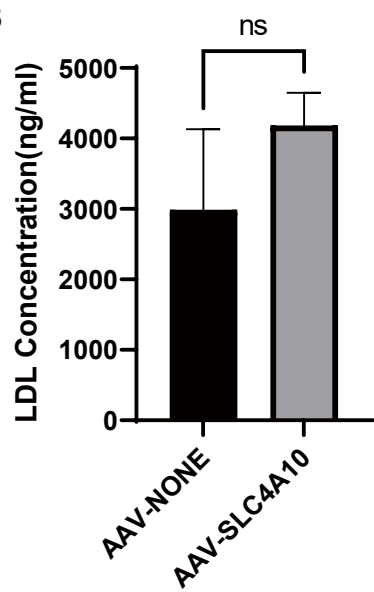

C

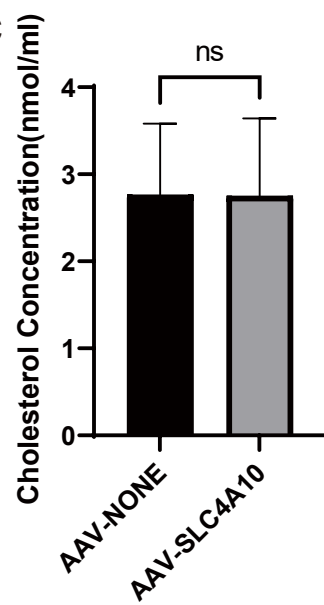

D

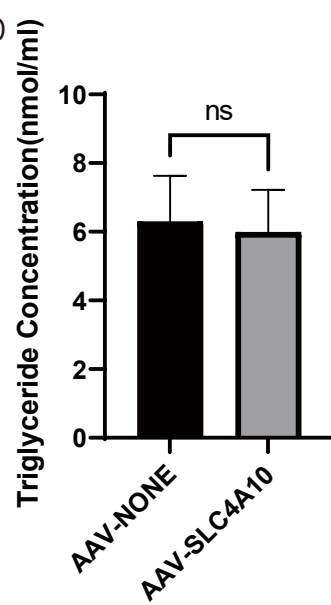

Supplement: Supplementary Figure 1 — Statistical analysis of serum HDL, LDL, cholesterol, and triglyceride levels in mice. [file Image1.pdf]

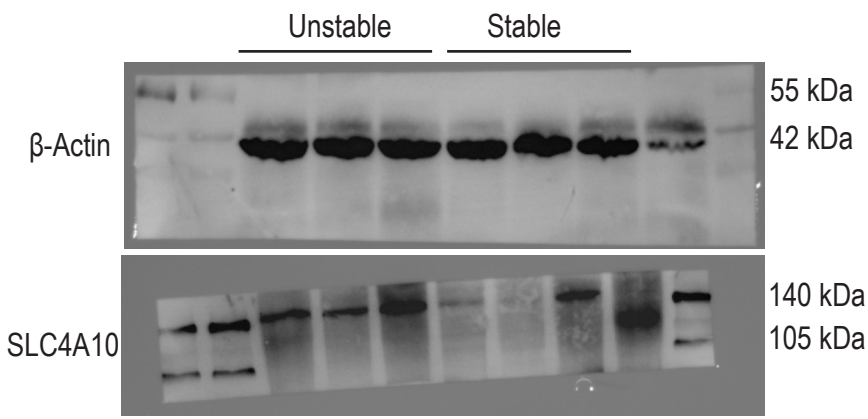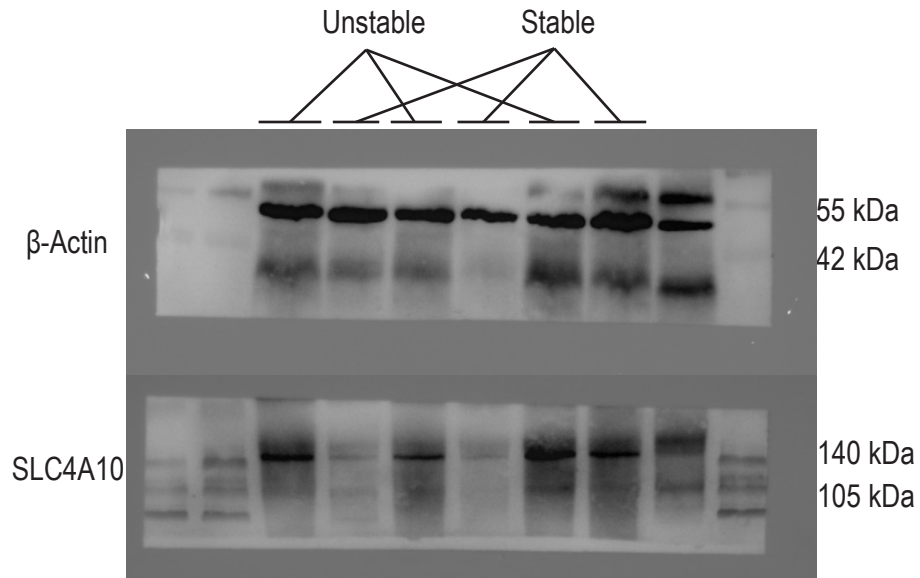

Supplement: Supplementary file 2 [file DataSheet1.zip › WB/Western blot image of human plaque.pdf]

AAV-NONE AAV-SLC4A10

$\beta$ -Actin

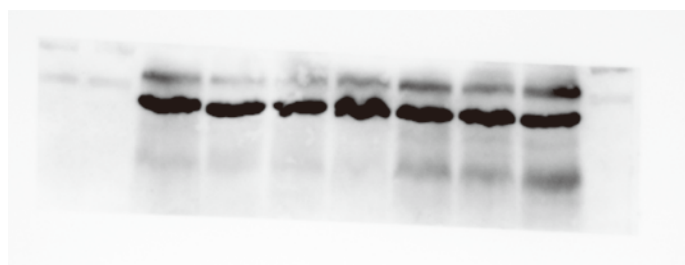

40 kDa

SLC4A10

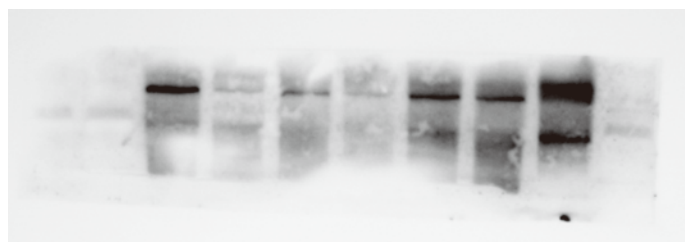

130 kDa

Supplement: Supplementary file 2 [file DataSheet1.zip › WB/Western blot image of mouse cells.pdf]

AAV-NONE   AAV-SLC4A10

$\beta$ -Actin

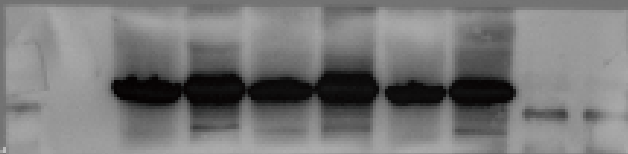

40 kDa

SLC4A10

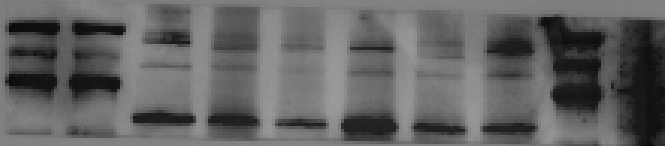

130 kDa

90 kDa

AAV-SLC4A10   AAV-NONE

$\beta$ -Actin

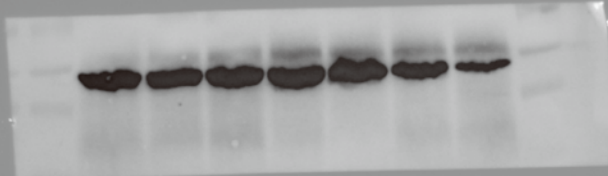

40 kDa

SLC4A10

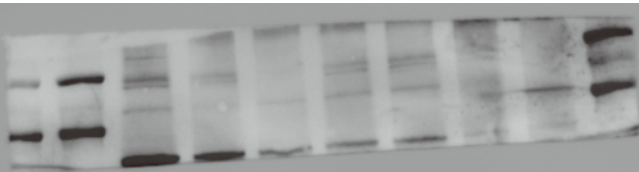

250 kDa

150 kDa

Supplement: Supplementary file 2 [file DataSheet1.zip › WB/Western blot of image mouse arteries.pdf]
