# Supplementary material for: SLC4A10 impedes atherosclerosis by diminishing IFN-γ/GZMB levels of CD8+ T cells via the MAPK pathway
Source: Front Immunol. 2025 May 29;16:1568999. doi: 10.3389/fimmu.2025.1568999 (PMC12159029; doi:10.3389/fimmu.2025.1568999)
Supplement: Supplementary file 2 [file DataSheet1.zip › gate strategy.pdf]

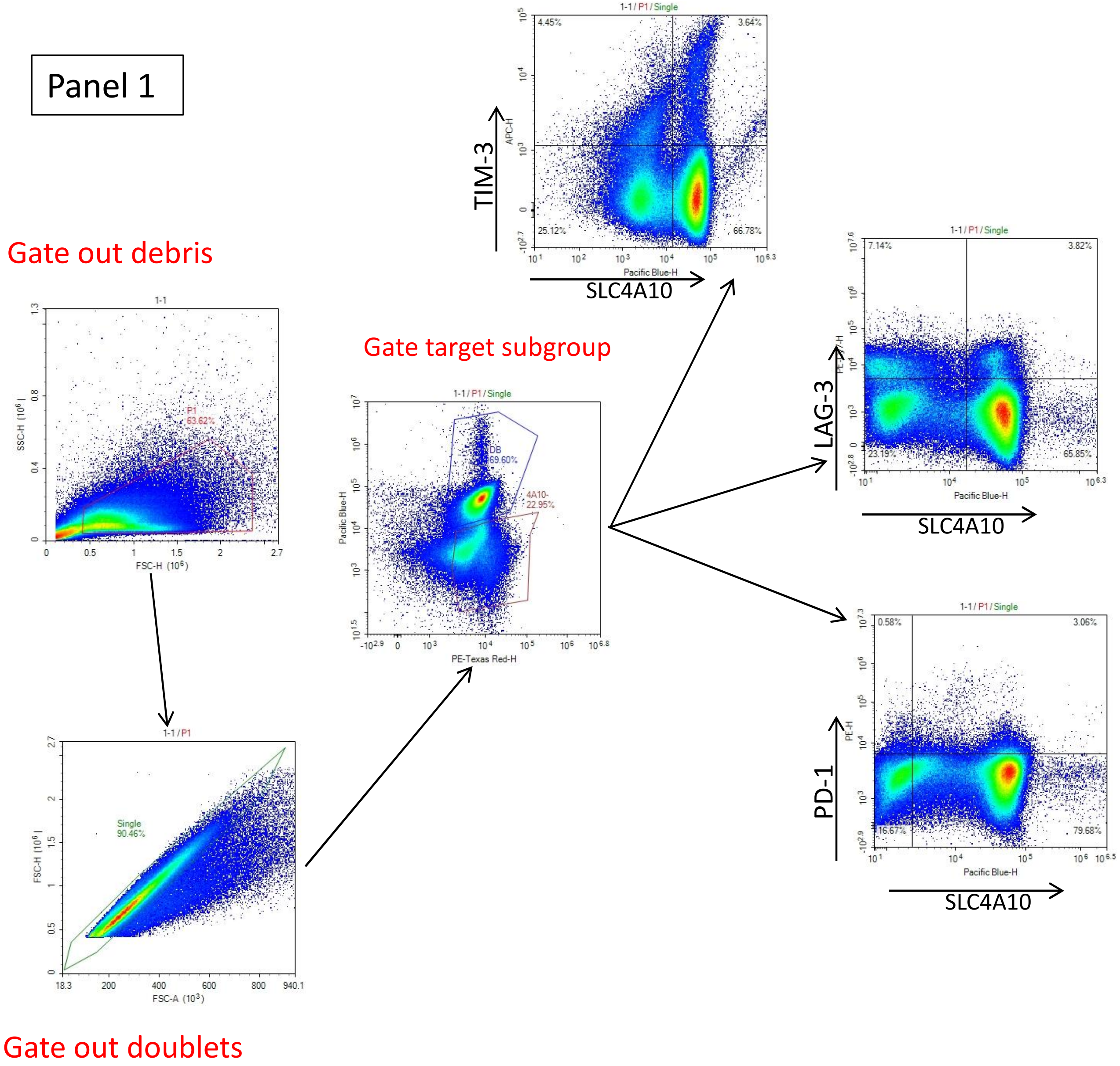
Panel 1
Gate out debris
1-1
P1
83.62%
SSC-H (10⁶)
FSC-H (10⁶)
Gate out doublets
1-1 / P1
Single
90.46%
FSC-H (10⁶)
FSC-A (10³)
Gate target subgroup
1-1 / P1 / Single
DB
69.60%
4A10-
22.95%
Pacific Blue-H
PE-Texas Red-H
TIM-3
APC-H
4.45%
3.64%
25.12%
66.78%
Pacific Blue-H
SLC4A10
LAG-3
7.14%
3.82%
23.19%
65.85%
Pacific Blue-H
SLC4A10
PD-1
PE-H
0.58%
3.06%
16.67%
79.68%
Pacific Blue-H
SLC4A10

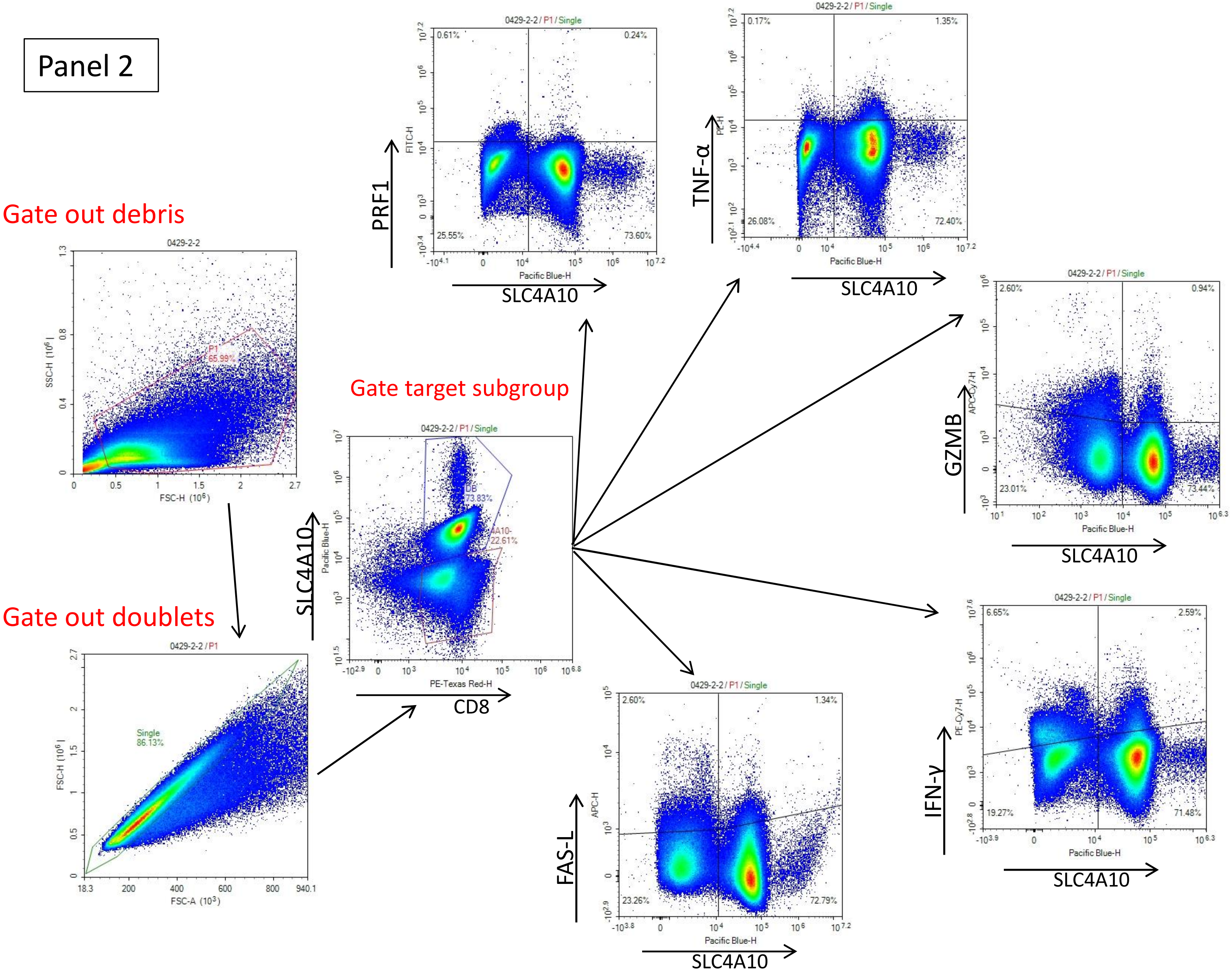
Panel 2
Gate out debris
0429-2-2
P1
65.99%
SSC-H (10⁶)
FSC-H (10⁶)
Gate out doublets
0429-2-2 / P1
Single
86.13%
FSC-H (10⁶)
FSC-A (10³)
Gate target subgroup
0429-2-2 / P1 / Single
DB
73.83%
A10-
22.61%
Pacific Blue-H
PE-Texas Red-H
SLC4A10
CD8
0429-2-2 / P1 / Single
0.61%
0.24%
25.55%
73.60%
FITC-H
Pacific Blue-H
PRF1
SLC4A10
0429-2-2 / P1 / Single
0.17%
1.35%
26.08%
72.40%
PE-H
Pacific Blue-H
TNF-α
SLC4A10
0429-2-2 / P1 / Single
2.60%
0.94%
23.01%
73.44%
APC-Cy7-H
Pacific Blue-H
GZMB
SLC4A10
0429-2-2 / P1 / Single
2.60%
1.34%
23.26%
72.79%
APC-H
Pacific Blue-H
FAS-L
SLC4A10
0429-2-2 / P1 / Single
6.65%
2.59%
19.27%
71.48%
PE-Cy7-H
Pacific Blue-H
IFN-γ
SLC4A10
