# Supplementary material for: SLC4A10 impedes atherosclerosis by diminishing IFN-γ/GZMB levels of CD8+ T cells via the MAPK pathway
Source: Front Immunol. 2025 May 29;16:1568999. doi: 10.3389/fimmu.2025.1568999 (PMC12159029; doi:10.3389/fimmu.2025.1568999)
Supplement: Supplementary file 3 [file Table1.docx]

**Supplementary Table 1: Antibodies**

| Antibody | Catalog number | Catalog numberVendor |
| --- | --- | --- |
| Alexa Fluor 700 Granzyme B | 372221 | BioLegend |
| FITC Perforin | 154309 | BioLegend |
| PE anti-mouse TNF-a | 506305 | BioLegend |
| APC FasL | 106609 | BioLegend |
| PE/Cvanine7 LAG-3 | 125225 | BioLegend |
| ABflo® 594 CD8a | A23905 | ABclonal |
| ABflo® 647 TIM-3 | A22779 | ABclonal |
| PE/Cyanine7 IFN-γ | 505825 | BioLegend |
| PE PD-1 | PE-65142 | Proteintech |
| Rabbit polyclonal anti-SLC4A8/10 antibody | TA315654 | OriGene |
| Sheep anti-rabbit IgG | A0208 | Beyotime |
| Brilliant Violet 421m Donkey anti-rabbit lgG | 406410 | BioLegend |
