# Supplementary material for: SLC4A10 impedes atherosclerosis by diminishing IFN-γ/GZMB levels of CD8+ T cells via the MAPK pathway
Source: Front Immunol. 2025 May 29;16:1568999. doi: 10.3389/fimmu.2025.1568999 (PMC12159029; doi:10.3389/fimmu.2025.1568999)
Supplement: Supplementary file 4 [file Table2.docx]

**Supplementary Table 2: Primer Sequences**

| Species | Gene Name | Primer Sequence |
| --- | --- | --- |
| Human | SLC4A10 | F:GTCACAGGCATCGTGGTCATA  R:CCTTCACGCCAACAAATCTCAT |
| Human | β-actin | F:CATGTACGTTGCTATCCAGGC  R:CTCCTTAATGTCACGCACGAT |
| Mouse | SLC4A10 | F:GTGGAACACGCTCTATTCTCAA  R:CCTCCAGTCCCGAATCTCTCT |
| Mouse | β-actin | F:GTGACGTTGACATCCGTAAAGA  R:GCCGGACTCATCGTACTCC |
| Mouse | GZMB | F:CCACTCTCGACCCTACATGG  R:GGCCCCCAAAGTGACATTTATT |
| Mouse | IFN-γ | F:CACAGGTCCAGCGCCAAGCA  R:CCCACCCCGAATCAGCAGCG |
| Mouse | TNF-α | F:TCCTGGCCAACGGCATGGAT  R:AATCGGCTGACGGTGTGGGT |
| Mouse | PD-1 | F:CAGCTTGTCCAACTGGTCG  R:GCTCAAACCATTACAGAAGGCG |
| Mouse | MAP2K6 | F:ATGTCTCAGTCGAAAGGCAAG  R:TTGGAGTCTAAATCCCGAGGC |
| Mouse | MAP3K5 | F:ACTCCCGGACCTTCATCACTA  R:ATGCCGCACTTTCTCTACCAC |
| Mouse | ELK4 | F:AGAATGAGCACATGATCTGCTG  R:AGGCTTGTTCTTGCGAATCCC |
